# Supplementary material for: Beyond Lux: methods for species and photoreceptor-specific quantification of ambient light for mammals
Source: BMC Biol. 2024 Nov 14;22:257. doi: 10.1186/s12915-024-02038-1 (PMC11562817; doi:10.1186/s12915-024-02038-1)
Supplement: Supplementary file 2 — Additional file 2: Figure S1. Spectral transmission for cornea, lens and vitreous humour for seven mammalian species. [file 12915_2024_2038_MOESM2_ESM.pdf]

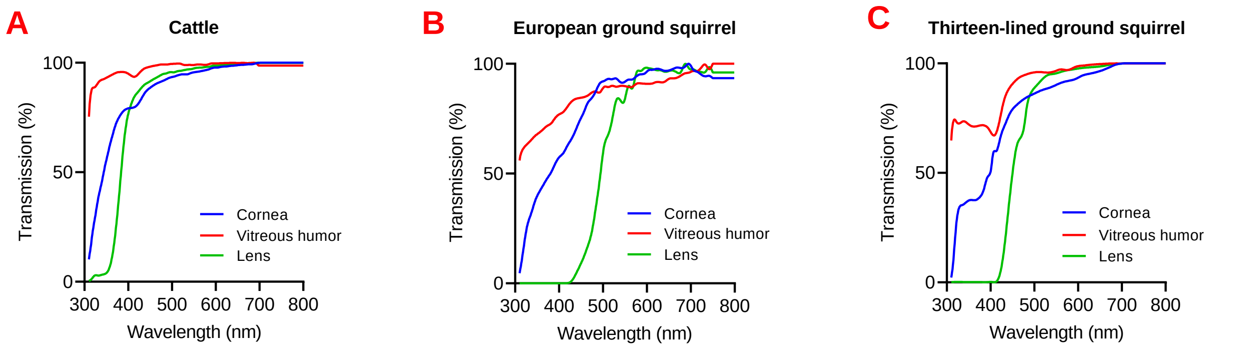

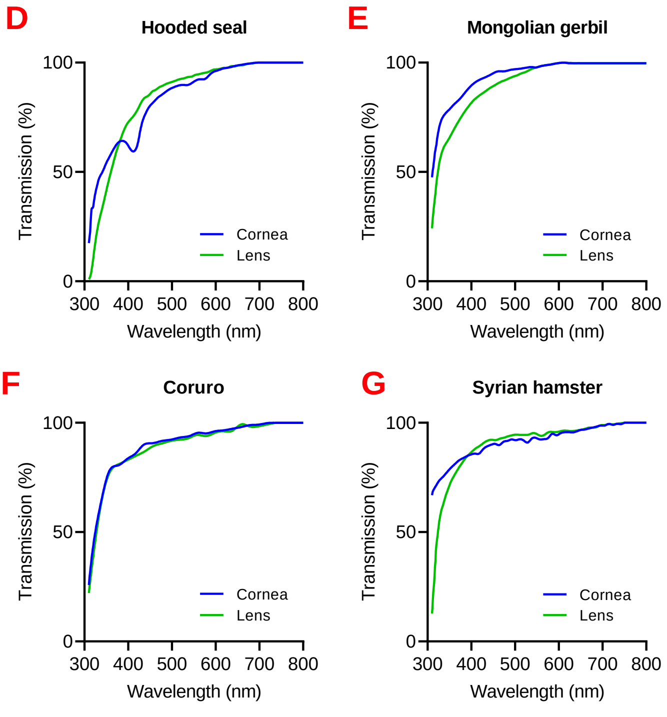

H

Wavelength at 50% transmission

**Species**	**Vitreous**	**Cornea**	**Lens**
Cattle	< 310	344	384
European ground squirrel	< 310	380	494
Thirteen-lined ground squirrel	< 310	399	450
Hooded seal	———	341	361
Mongolian gerbil	———	311	324
Curoro	———	325	328
Syrian hamster	———	< 310	324
