# Supplementary figures and images for: Beyond Lux: methods for species and photoreceptor-specific quantification of ambient light for mammals
Source: BMC Biol. 2024 Nov 14;22:257. doi: 10.1186/s12915-024-02038-1 (PMC11562817; doi:10.1186/s12915-024-02038-1)

**A****i)**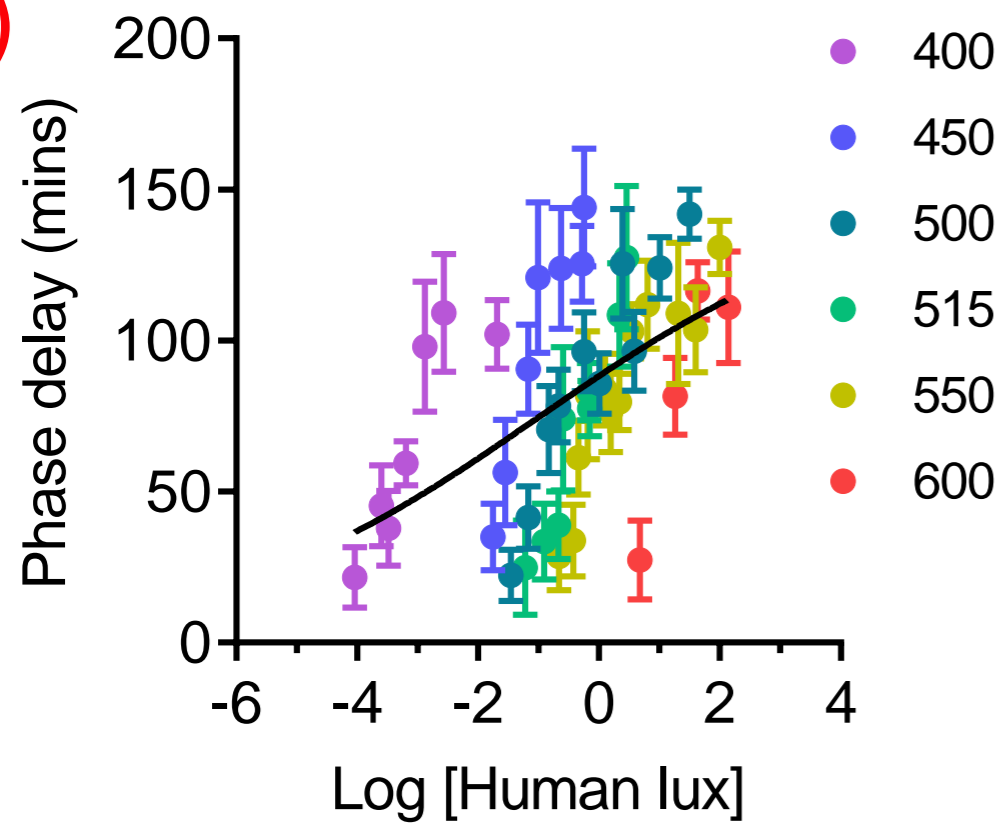**ii)**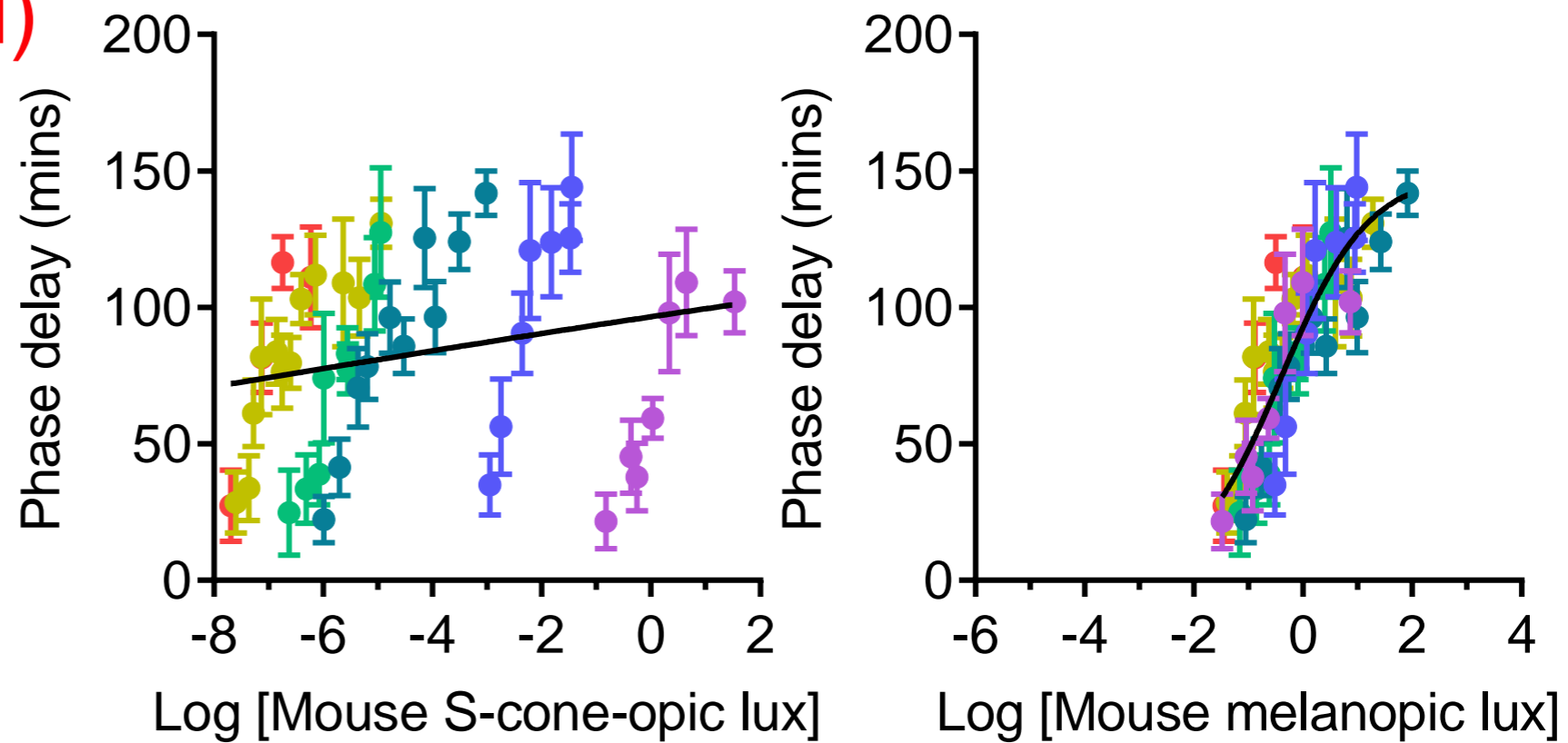**iii)**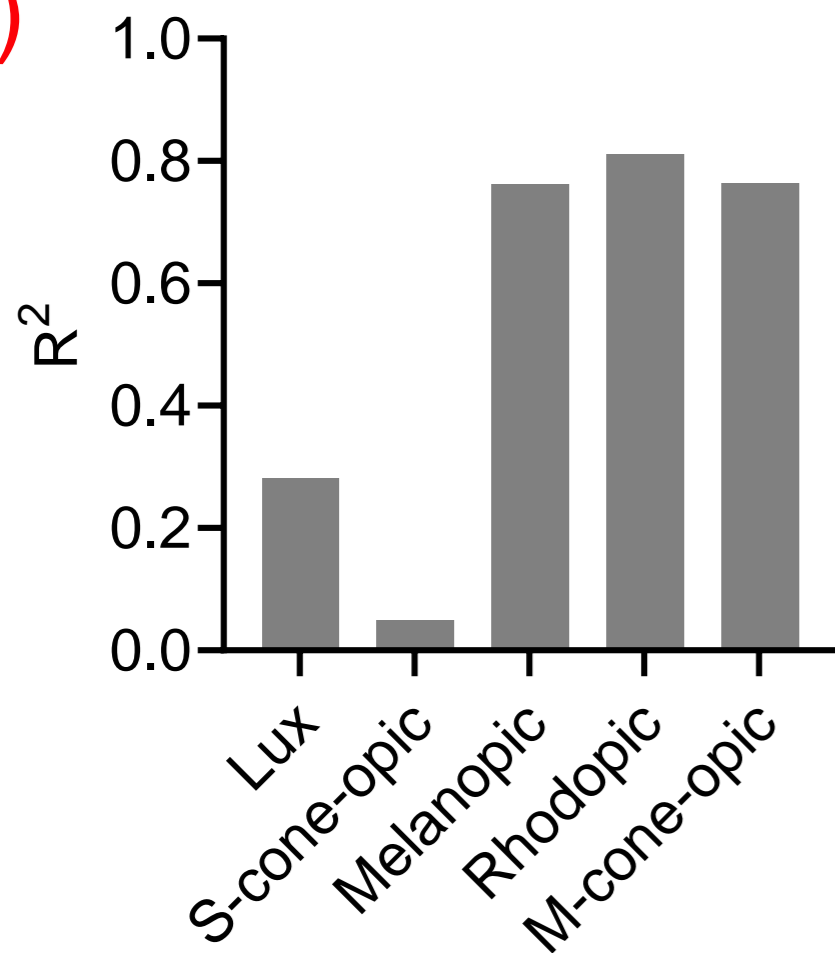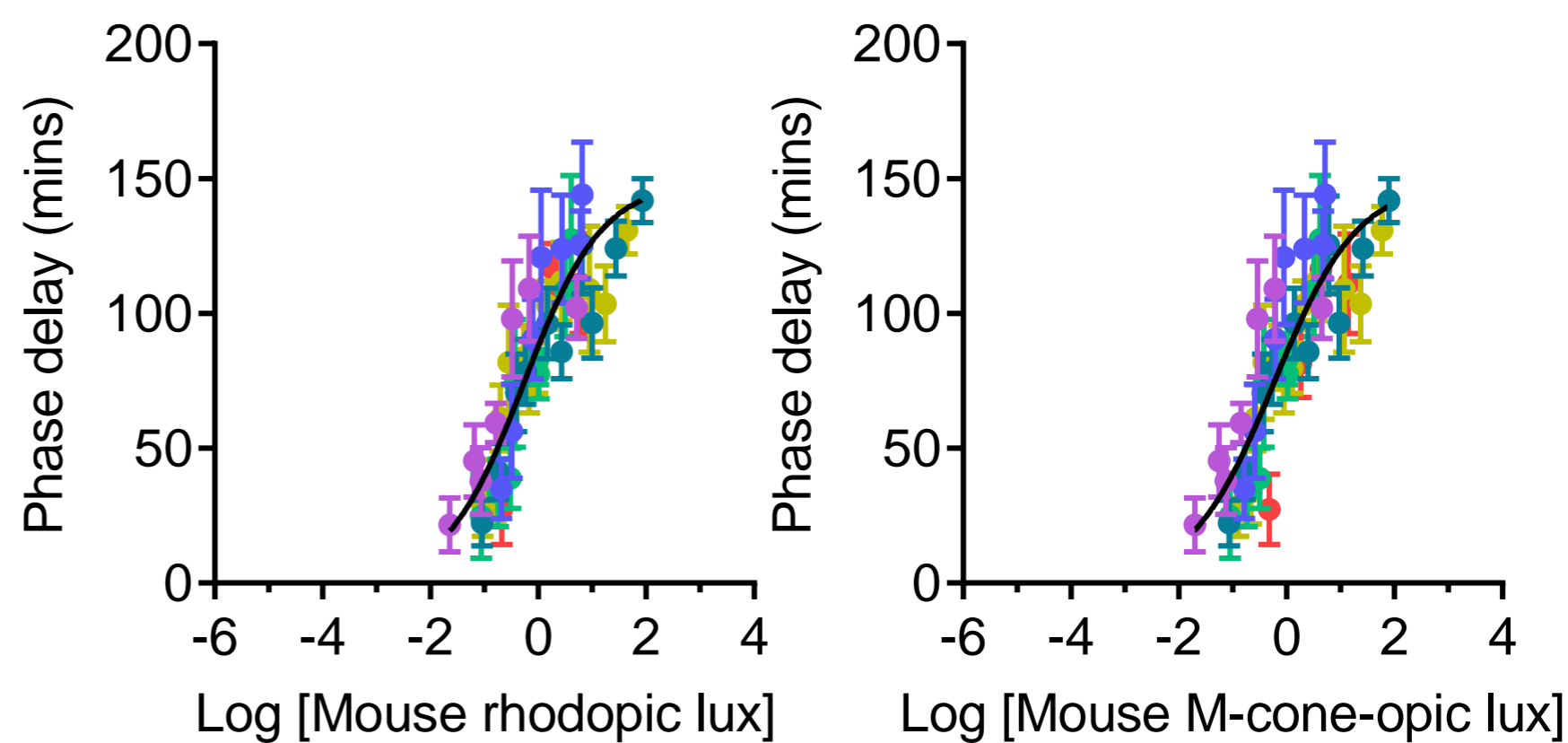

**B****i)**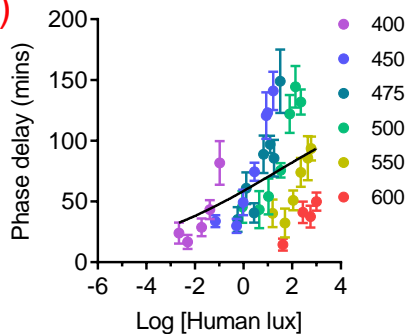**ii)**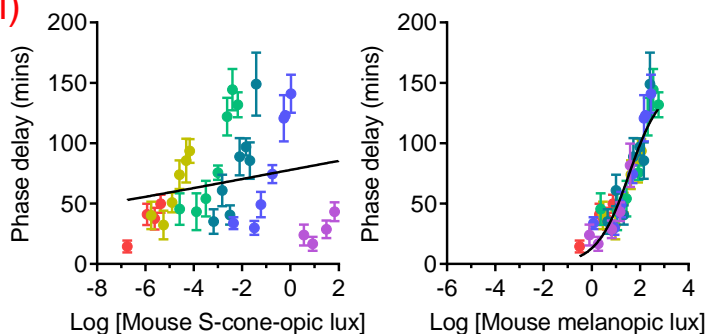**iii)**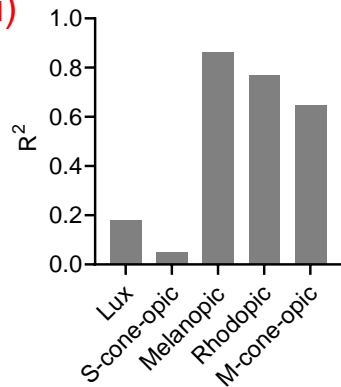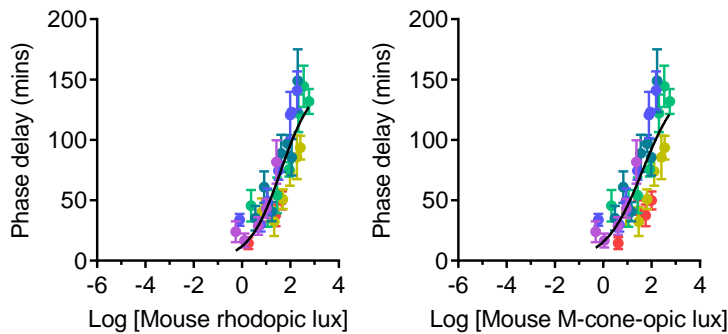

**C****i)**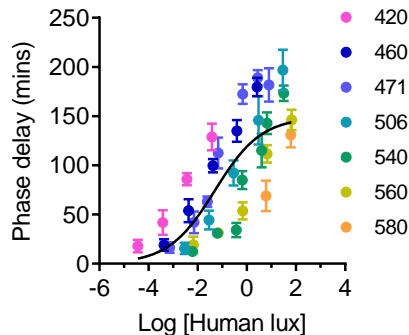**ii)**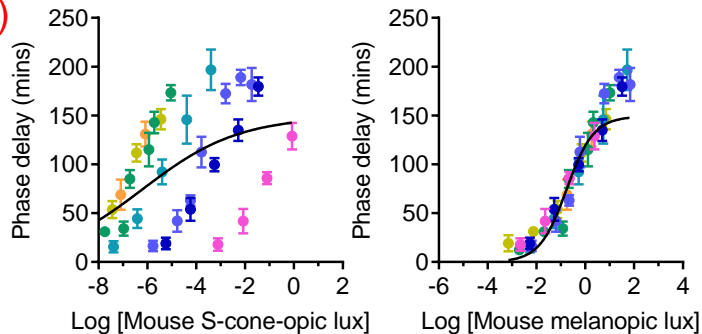**iii)**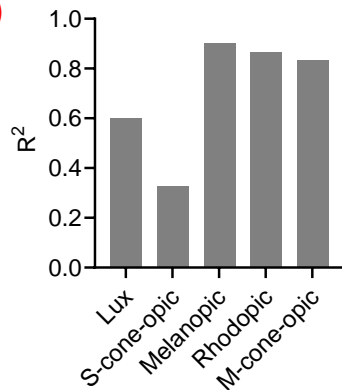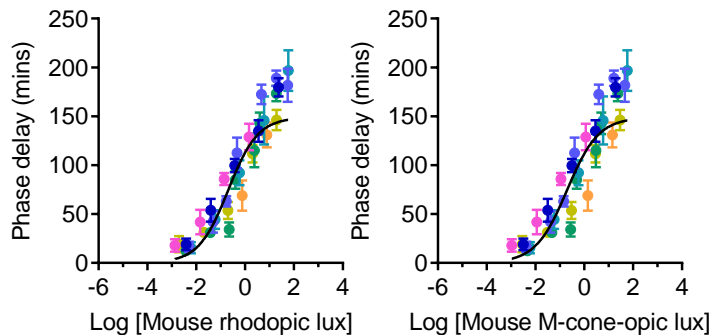

Supplement: Supplementary file 5 — Additional File 5: Figure S2. Irradiance response curves for circadian phase shifts in wild-type and retinally degenerate mice expressed as a function of photopic lux or mouse α-opic EDIs. Figure S2A: Irradiance response curves for circadian phase shifting in CBA/N (+/+) mice mouse [45]. Figure S2B: Irradiance response curves for circadian phase shifting in retinally degenerate CBA/J (rd/rd) mouse [45]. Figure S2C: Irradiance response curves for circadian phase shifting in retinally degenerate rd/rd cl mouse [47]. [file 12915_2024_2038_MOESM5_ESM.pdf]
